# Moving together - benefits of an online dance program on physical and mental health for older women: an exploratory mixed-method study

**DOI:** 10.1186/s12877-024-04983-6

**Published:** 2024-05-02

**Authors:** Rasmus Kopp Hansen, Elizabeth Jochum, Ditte Egholm, Morten Villumsen, Rogerio Pessoto Hirata

**Affiliations:** 1 ExerciseTech, Department of Health Science and Technology, Aalborg University, Selma Lagerløfs Vej 249, Aalborg, DK-9260 Gistrup Denmark; 2 Department of Communication and Psychology, Aalborg University, Aalborg, Denmark; 3 Center for Orthopedic Rehabilitation and Head of Center for Fall Prevention, Marselisborg Rehabilitation Center, Aarhus Municipality, Aarhus, Denmark; 4 Department of Health Science and Technology, Faculty of Medicine – Pain and Motor System Plasticity, Aalborg University, Aalborg, Denmark; 5 Respiratory and Critical Care Group, Department of Health Science and Technology, Aalborg University, Aalborg, Denmark

**Keywords:** Older adults, Physical activity, Dance, Community implementation, Mental health, Physical health

## Abstract

**Background:**

Previous studies show that in-person dance training is a beneficial form of physical activity that involves mental, social, and physical dimensions. This exploratory study investigated the benefits of a 12-week online dance training intervention on mental and physical health outcomes for older women.

**Methods:**

A convergent parallel mixed-method design was used. Forty-five older adults (74.0 ± 5.3 yrs old, 44 women) were recruited through advertisements at activity and rehabilitation centers in the North Denmark region. The intervention consisted of two weekly 60-min classes of improvisation and salsa delivered online through video call applications. Changes in physical health outcomes (body mass and composition, resting blood pressure, Senior Fitness Test battery) and self-rated health and wellbeing (health-related quality of life (HRQOL), feelings of loneliness) were assessed prior to and after 12 weeks of dancing. Focus group interviews were conducted post-intervention to further explore the benefits as well as the participant’s experience of the intervention. Thematic analysis of the qualitative data was conducted.

**Results:**

Thirty-two participants (all women) completed the study. Significant improvements in fitness were found for the number of arm curls performed (baseline: 12.3 ± 3.0; post-intervention: 13.7 ± 3.0, *P* = 0.005), 2-min step test performance (baseline: 66.5 ± 20.0 reps.; post-intervention: 73.8 ± 22.6 reps., *P* = 0.016), and chair sit-and-reach (baseline: 0.4 ± 11.3 cm; post-intervention: 5.5 ± 10.1 cm, *P* < 0.001). There was a significant increase in body mass from baseline to post-intervention (*P* < 0.015). The themes from the focus groups included (1) Participation, (2) Challenges, (3) Progression, (4) Motivation, (5) Perceived health and wellbeing, and (6) Online dance instruction. No significant changes were reported in HRQOL and loneliness from the quantitative data, although the qualitative data did reveal improved feelings of physical health and wellbeing.

**Conclusions:**

The intervention improved several aspects of fitness in older women and improved the participants’ perceptions of their own physical abilities and wellbeing. While most participants found the online intervention enjoyable, several participants missed the feedback from the instructors that naturally occurs with in-person instruction.

## Introduction

Physical inactivity is commonly observed among older adults (65 years of age or older) [1]. In addition to elevated mortality risk [2], physical inactivity also presents increased risks of social isolation and loneliness, which are known to be major risk factors for negative health outcomes, such as reduced health-related quality of life (HRQOL) [3, 4], particularly among older adults [5]. Although many older adults have positive attitudes toward physical activity, there are many factors that limit their participation, including safety concerns, low self-efficacy, pre-existing medical conditions, low physical fitness, time constraints, transportation, and culture [6].

Previous research has shown that arts and arts-based activities can positively impact physical and mental health outcomes by fostering creativity and promoting meaningful social engagement, both of which can improve HRQOL [7]. More specifically, unlike the majority of other types of physical activity, dance is an aesthetic form that allows for creative expression and is socially engaging [8]. Dancing can result in physical benefits comparable with those of formal exercise training [9], and has been found to improve social engagement and HRQOL [7, 10, 11]. In addition, Keogh and colleagues found that dance improved the emotional, psychological, and physical well-being of individuals [12], and Ambegoankar et al. 2022 found that weekly dance sessions had a positive impact on physical health and cognition for community dwelling older adults [7]. Comprehensive assessment of dance activities and movement-based therapies frequently assess improvement according to physiological benchmarks [12]. However, apart from the above examples, there are scant studies that fully consider the combined physical, psychological, and social impacts of dance.

Following extensive periods of social isolation, online interventions have proven to be an effective first-step for returning to physical activity [13], and for improving functional fitness and mental health [14]. Online interventions can facilitate social support and connectivity by connecting like-minded individuals or groups who are interested in becoming more physically active [13]. Social support can provide individuals with motivation, accountability, and a sense of belonging, which can help them stay on track with their physical activity goals [15] and support independent lifestyles and healthy aging [7]. Furthermore, online interventions allow more flexibility in terms of when and where to engage in physical activity [14], factors that are especially important for individuals who may not have access to traditional exercise facilities and would like to exercise from home [13], or who otherwise face challenges leaving their home.

Dance is a complex intervention and thus requires complex methods for ensuring that the effects of dance training are fully understood from a holistic point of view. Measuring the impact of online dance training on physical and mental outcomes thus requires a mixed-methods approach and insights from across scientific disciplines. Accordingly, the aim of the Moving Together project was to conduct a mixed-method evaluation to explore the benefits of a 12-week online dance training intervention on physical and mental health outcomes for older adults.

## Methods

### Study design

A mixed-method, pre-post study was designed to explore the influence of a 12-week online dance intervention on physical and mental health in older adults. This exploratory uncontrolled study used a convergent parallel mixed-method design, which involves collecting and analyzing quantitative and qualitative data concurrently but separately and merging the data at the interpretation stage to arrive at a comprehensive and holistic understanding of the data [16]. The Moving Together study builds upon a previous randomized controlled trial from our group [17] which demonstrated that regular circuit training and a combination of online and in-person dance training significantly reduced the number of fall accidents in older adults compared with the control group [18]. We sought to build on this work by focusing on implementing an online dance training program, and thus the study was conducted in close collaboration with regional activity and rehabilitation centers in the North Denmark region that streamed the online dance classes. The dance intervention was open for all interested older adults residing in the municipality for an eight-month period, however only a subgroup of these were included in the current study, based on a first-come/first-served basis. The duration of the study was from November 2022 to May 2023. The study was approved by the North Denmark Region Committee on Health Research Ethics (N-20220045) and conducted in accordance with the Declaration of Helsinki. Participants were recruited through advertisements at the activity and rehabilitation centers and with assistance from the municipality. After receiving oral and written information about the study, all participants provided written informed consent.

### Participants

To ensure inclusivity of participants, only two inclusion criteria were used: (a) being 65 yrs or older, and (b) being able to speak and understand Danish. Exclusion criteria included drug addiction, being unable to stand and walk independently, lack of ability to cooperate, and participation in medical trials or other training intervention studies in parallel. No criteria were used in terms of prior dance experience. Because of the broad inclusion criteria, there was a wide range in both age (65 to 87 yrs) and the physical capacity of the study participants.

A total of 45 participants (44 women) were included in the study (Table 1). This number was based on a sample size calculation using a paired t-test (two-tailed). With a statistical power of 0.9, an alpha level of 0.05, and an assumed moderate effect size (d = 0.55) in mental health outcome, as previously demonstrated after Dance Movement Therapy [19], 37 participants were required to detect a significant change in self-reported loneliness (a main indicator of mental health and social wellbeing). To account for dropouts during the 12-week intervention period (estimated 20%), 45 participants were included.

**Table 1:** Baseline participant characteristics

	Online dance intervention (*N* = 45)	
Sex, W/M	44/1	
Age, yr	74.0 ± 5.3	
Height, m	1.62 ± 0.58	
Body mass, kg	69.7 ± 13.0	
BMI, kg/m^2^	26.4 ± 4.9	
SBP, mmHg DBP, mmHg	133 ± 15 79 ± 8	

W = Women; M = Men; BMI = Body mass index; SBP = Systolic blood pressure; DBP = Diastolic blood pressure. Values are n, or mean ± SD

### Description of intervention

The dance intervention consisted of 12 weeks of two weekly classes of 60 min training in ‘improvisation’ (contemporary dance) (Day 1, Tuesday) and Cuban salsa (Day 2, Thursday), led by professional dance instructors experienced with online teaching and trained in inclusive dance practice. The attention to inclusive practice was necessary given the broad inclusion criteria, and modifications were offered throughout the study to meet the different experience levels and physical abilities of the study participants. The improvisation classes were structured around a gradual increase in intensity through the one hour of practice. The movement language of Laban’s choreological practice [20] was used to inspire and create variations of movement. The classes both started and finished with a focus on breathing exercises. The salsa classes were based on Cuban salsa and included a gradual introduction to the technique and the expressive aesthetics of the dance style. In time, the class included more improvised salsa and dancing at a faster pace. No baseline assessment was performed of how familiar participants were with the two types of dances. Both classes were delivered online through video calls using the Zoom meeting application (Zoom Video Communications, Inc., San Jose, CA, USA), with remote instruction by the dance instructors. The classes followed a similar structure and lasted one hour, which included a warmup, main session, and a cool down. To allow flexibility, participants could choose to attend the online classes from home (alone) or at one of four activity centers in the municipality (group-based). Recordings of the dance instructor were made at each online class and uploaded to a designated YouTube channel created specifically for the project [21]. The YouTube channel was a resource made available for all participants, and anyone else interested, where participants could review and replay the classes if they were unable to attend a scheduled class. Participants were instructed to follow the dance classes (live or asynchronous) at least once a week but were strongly encouraged to dance twice weekly. To promote engagement and raise awareness of the intervention, a 60-min in-person salsa dance class followed by lunch was conducted once every month with physical attendance by both the instructor and the participants (up to three sessions in total per participant). Besides this monthly in-person dance class, no further social activities were encouraged as part of the study. Given the exploratory uncontrolled study design and focus on implementation in practice, no specific restrictions were made on the participants regarding participation in other sports or training activities during the study period.

### Quantitative outcomes

All outcome measurements (except focus group interviews; only post-intervention) were performed immediately prior to (baseline) and after the 12-week intervention period (post-intervention) and conducted at activity centers in the municipality (physical assessment and questionnaires) or online (interviews).

#### Resting blood pressure

After at least five min of rest, systolic blood pressure and diastolic blood pressure were measured with the participants comfortably seated in a chair with uncrossed legs using an automated monitoring device (OMRON M3, OMRON Healthcare, Hoofddorp, The Netherlands). Measurements were performed in duplicate, with a third measurement performed if the measurements deviated by > 5%. The lowest values were used for further analyses.

#### Body composition and anthropometric assessments

Body mass and height were measured with the participants wearing light clothing, without shoes and rounded to the nearest 0.1 kg and 0.5 cm, respectively. BMI was calculated as body mass divided by height squared (kg/m^2^). Waist circumference was measured at the midpoint between the top of the iliac crest and the lower margin of the last palpable rib with a non-elastic tape measure, standing with the feet together and arms down following a normal expiration [22]. Hip circumference was measured around the greater trochanter [22]. Waist-hip ratio was calculated as waist circumference divided by hip circumference. For both waist and hip circumference, measurements were performed in duplicate, and reported as the mean. If the difference between the first and second measure was > 0.5 cm, a third measure was obtained. Skinfold thickness was measured at four standard sites (biceps, triceps, subscapular, and suprailiac) using a Harpenden Skinfold Caliper. Skinfold thickness was then converted to body density using Durnin and Wormersleys linear regression equation, used in the Siri Equation for estimation of body fat percentage [23]. The same experienced investigator performed all waist and hip circumference and skinfold measurements and was blinded at the post-intervention assessment to the results obtained at the baseline visit.

#### Senior fitness test

The Senior Fitness Test (SFT) battery was used to assess functional fitness, which can be defined as the capacity to perform usual everyday activities safely and independently [24]. The SFT has shown high content and criterion validity and consists of six field-based test items to evaluate upper and lower body muscle strength and flexibility, aerobic endurance, and dynamic balance/agility [25]. The SFT is comprised of a 30-second chair stand test and arm curl (that assess lower and upper body strength, respectively), a 2-min step test (evaluating aerobic endurance), chair sit-and-reach and back scratch (assessing hamstring and shoulder flexibility, respectively), and 8-foot-up-and-go (evaluating dynamic gait/agility) [26]. The procedures for administering the SFT were standardized as described in detail by [25]. Scoring for each item was performed on a continuous scale, with no total score. For five of the items, the higher the score the better, whereas for one item (the 8-foot-up-and-go), the lower (i.e., faster) the score the better.

#### Self-rated health and wellbeing

The Short-Form Health Survey 36 (SF-36) and the University of California, Los Angeles (UCLA) Loneliness Index were used to assess participants perceived mental health and wellbeing. The Danish translated version [27] of the SF-36 was completed for assessment of self-reported HRQOL [28]. Data was scored using the RAND 36-item Health Survey (version 1.0) method [29]. Pre-coded numeric values for each of the 36 questions were transformed into a score from 0 to 100, with a higher score representing a more favorable health status. Questions belonging to the same subscale were then averaged together to create eight health summary scores (four representing mental quality of life (QoL) and four representing physical QoL) [28]. Loneliness and social isolation were evaluated through the Danish translated version [30] of the UCLA Loneliness Scale version 3 [31]. A total of 20 questions were answered on a 4-point Likert scale ranging from 1 (Never) to 4 (Often). Based on the scoring of each of the 20 items, a Total Loneliness Score (TLS) was calculated, ranging from 20 to 80, with higher scores indicating greater degrees of loneliness. The Danish translated version of the UCLA loneliness has good psychometric properties that support the reliability and validity of the scale [30]. For descriptive purposes, the extent of loneliness was categorized as follows: 20–34 (low degree of loneliness); 35–49 (moderate degree of loneliness); 50–64 (moderately-high degree of loneliness); and 65–80 (high degree of loneliness) [32].

#### Qualitative outcomes

Focus group interviews were conducted post-intervention and following the post-physical assessments. All participants, including those who did not complete the intervention, were invited for interviews. The interviews were performed online via the Zoom meeting application, and conducted by the same experienced interviewer who was also one of the interventionists and a member of the research team. The interviews contained questions about the participants’ experiences of the intervention, self-perception of impact on physical and mental health, including possible effects of the intervention in other areas of their life, such as activities of daily living (ADL). Participants were also prompted to give qualitative feedback on the instruction and the experience of participating in online classes.

### Data analysis

#### Quantitative analysis

Normal distribution of data was confirmed with the Shapiro-Wilk Test. Standardized effect sizes (Cohen d) were calculated to determine the magnitude of change (pre-post), with the following thresholds: trivial (< 0.2), small (≥ 0.2), moderate (> 0.5), and large (> 0.8). Before and after comparisons on outcomes were performed using two-sided student’s paired-t tests. Statistical analyses were performed using SPSS (version 27; IBM, Armonk, New York). Statistical significance was accepted at alpha < 0.05. In the text and tables, values are means with SD, unless otherwise stated.

#### Qualitative analysis

A total of eight focus groups were conducted with two to four participants per focus group. Participants were given the option either to take part in the interview from their home or in a room at the activity center and were interviewed by a member of the research team through Zoom. The interviews lasted approximately 60 min; audio recordings were made and transcribed using an automatic transcription tool (Mygoodtape.com) and following manual corrections and were manually reviewed by the interviewer and corrected for typographical mistakes or spelling errors. The transcriptions were then translated into English by the interviewer (a native Danish speaker fluent in written and spoken English). Transcripts from the interviews were analyzed independently by two members of the research team: the interventionist, and a researcher familiar with the study but not present at the interviews. The two researchers conducted thematic analysis of the textual data separately, following the six general steps outlined by Braun and Clarke [33] using a combination of inductive and deductive approaches, as well as a combination of semantic and latent approaches. After the researchers completed this task independently, they met to discuss commonalities and differences and then repeated the steps together. Textual data was coded, and sub-themes and themes emerged (Table 2), which were then presented in plenum to the entire research team. The research team also followed Braun and Clark’s 15-point criteria to ensure sound research practice [33]. As the interview data was translated and coded in a different language than the interviews took place in, attention to these criteria was especially important during the transcription, coding, and analysis phases.

**Table 2:** Focus group main themes and subthemes identified through thematic analysis (focus groups, *N* = 8; participants, *N* = 28)

**Theme 1: Participation**	
Subtheme 1.1	Description of individual participation
Subtheme 1.2	Preferences for instruction
Subtheme 1.3	Level and quality of instruction
Subtheme 1.4	Obstacles to participation
**Theme 2: Challenges**	
Subtheme 2.1	Technical difficulties
Subtheme 2.2	Challenges with their own movement
Subtheme 2.3	Challenges with the instruction
Subtheme 2.4	Self-consciousness
Subtheme 2.5	Challenges with the method
**Theme 3: Progression**	
Subtheme 3.1	Physical progression
Subtheme 3.2	Skill transference
**Theme 4: Motivation**	
Subtheme 4.1	Social connection
Subtheme 4.2	Involvement in research study
Subtheme 4.3	Interest in dancing
**Theme 5: Perceived Health & Well-Being**	
Subtheme 5.1	Physical changes
Subtheme 5.2	Social, emotional, and mental health
Subtheme 5.3	Connection to everyday life
**Theme 6: Online Dancing**	
Subtheme 6.1	Familiarity / freedom of movement
Subtheme 6.2	Connection to instructors
Subtheme 6.3	Lack of feedback
Subtheme 6.4	Flexibility of YouTube
Subtheme 6.5	Individual effort
Subtheme 6.6	Suggestions for improvement

## Results

### Participants

Of the 45 participants included at baseline, 32 participants (all women) completed the study. All 32 participants were included for analyses irrespective of how they attended the dance classes (live or recorded class uploaded to YouTube) and where (at activity center or at home). Reasons for dropping out included non-study related health issues (*N* = 5); COVID-19 infection (*N* = 1); being the only man (*N* = 1); lack of motivation (*N* = 4); and finding it too physically demanding to complete the dance sessions (*N* = 2).

### Body composition, anthropometric, and resting blood pressures

There was a significant increase in BMI from baseline (26.4 ± 4.7 kg/m^2^) to post-intervention (26.7 ± 4.6 kg/m^2^, *P* = 0.016, d = 0.45). Similarly, there was an increase in body mass from baseline (69.4 ± 13.0 kg) to post-intervention (70.1 ± 12.7 kg, *P* = 0.015, d = 0.45). In contrast, there was no significant change in waist circumference from baseline (95.3 ± 11.6 cm) to post-intervention (94.0 ± 12.9 cm, *P* = 0.051, d = 0.36). There were no changes from baseline to post-intervention in sum-of-skinfolds (72.8 ± 22.4 mm vs. 74.2 ± 24.2 mm, *P* = 0.352, d = 0.17), body fat percentage (37.6 ± 4.4% vs. 37.8 ± 4.4%, *P* = 0.271, d = 0.20), or waist-hip-ratio (0.91 ± 0.05 vs. 0.90 ± 0.06, *P* = 0.613, d = 0.09). No changes were observed from baseline to post-intervention in systolic blood pressure (132 ± 15 mmHg vs. 132 ± 18 mmHg, *P* = 0.850, d = 0.03) or diastolic blood pressure (79 ± 9 mmHg vs. 79 ± 8 mmHg, *P* = 0.932, d = 0.02).

### Functional fitness (senior fitness test)

**Fig. 1:**
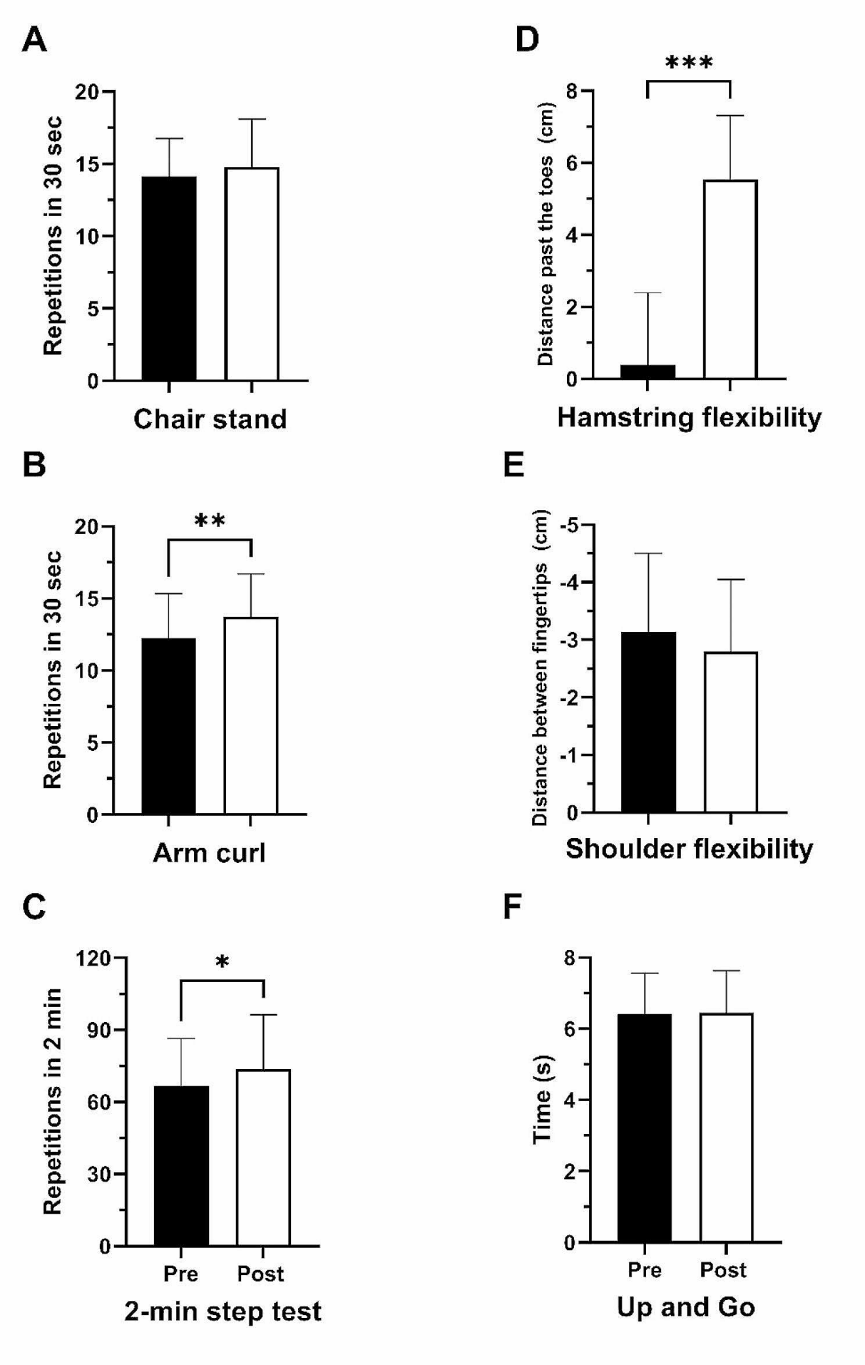
Changes in Senior Fitness Test items following 12 weeks of dance training (**A**), number of chair stands performed in 30 s, (**B**) number of arm curls performed in 30 s, (**C**) number of steps performed in 2 min, (**D**) fingertip distance behind (-) or past (+) the toes during chair sit-and-reach, (**E**) distance behind (-) or past (+) fingertip to fingertip during back scratch, (**F**) time in seconds to complete the 8-foot Up-and-Go. Data are means ± SD, except for panel D and E where error bars represent SEM. ***P < 0.001, **P < 0.01, *P < 0.05

Data from the SFT battery are shown in Fig. 1. Significant improvements in fitness from baseline to post-intervention were found for the number of arm curls performed (baseline: 12.3 ± 3.0; post-intervention: 13.7 ± 3.0, *P* = 0.005, d = 0.54, Fig. 1B), 2-min step test performance (baseline: 66.5 ± 20.0 reps.; post-intervention: 73.8 ± 22.6 reps., *P* = 0.016, d = 0.45, Fig. 1C), and chair sit-and-reach (baseline: 0.4 ± 11.3 cm; post-intervention: 5.5 ± 10.1 cm, *P* < 0.001, d = 1.05, Fig. 1D). There were no significant changes in the number of chair stands performed (*P* = 0.143, d = 0.27), shoulder flexibility (*P* = 0.630, d = 0.09), or in the time to complete the 8-foot Up and Go (*P* = 0.697, d = 0.07).

### Self-rated health and wellbeing

Results for total loneliness score (TLS) and HRQOL summary scores are shown in Table 3. There was no significant change in TLS from baseline to post-intervention (*P* = 0.054, d = 0.36). There were no significant changes in any of the eight HRQOL summary scores (all *P* ≥ 0.118) with small effect sizes (d ≤ 0.28).

**Table 3:** Self-reported loneliness and health-related quality of life at baseline and after 12 weeks of online dance (*N* = 32)

	Baseline	Post-intervention	*P*-value	Effect size (d)
**Loneliness (TLS)**	35.5 ± 7.1	33.5 ± 7.0	0.054	0.36
**Degree of loneliness**				
Low (20–34)	15 (47%)	18 (56%)		
Moderate (35–49)	17 (53%)	14 (44%)		
Moderate-high (50–64)	0 (0%)	0 (0%)		
High degree (65–80)	0 (0%)	0 (0%)		
**HRQOL ** **Physical health**				
Physical functioning	85.2 ± 16.0	85.9 ± 12.9	0.633	0.09
Role-Physical	82.8 ± 31.4	82.0 ± 30.6	0.879	0.03
Bodily pain	85.2 ± 19.0	86.6 ± 14.1	0.551	0.11
General health	77.7 ± 14.4	76.6 ± 14.5	0.699	0.07
**HRQOL** **Mental health**				
Vitality	75.6 ± 15.2	76.6 ± 13.6	0.764	0.05
Social functioning	95.3 ± 9.9	93.8 ± 14.9	0.619	0.09
Role-Emotional	94.8 ± 12.3	86.5 ± 27.9	0.118	0.28
Mental health	86.1 ± 12.9	85.6 ± 11.6	0.854	0.03

Values are n (%) or mean ± SD

### Qualitative responses

Of the 45 study participants invited to the interviews, 28 took part (various reasons were given for declining, mostly schedule conflicts or illness). Six main themes were identified: (1) Participation, (2) Challenges, (3) Progression, (4) Motivation, (5) Perceived health and wellbeing, and (6) Online dance instruction. Table 2 presents the main themes and subthemes identified through thematic analysis.

### Participation

Within this main theme, four subthemes were identified: description of individual participation, preferences for instruction, level and quality of instruction, and obstacles to participation. The subthemes reflect variety of ways that study participants received the instruction: the majority chose to participate in-person at one of the activity centers, and only a few participants reported training alone at home. The subthemes also reflect the variety of ways the online classes were screened. Depending on the activity center, online classes were either streamed live (where the instructor could see and interact with the participants at home and the activity center), or else they were streamed from pre-recorded sessions available on the YouTube channel (no interaction between instructor and participants). One center streamed only the salsa classes, and another used an on-site physiotherapist to guide and facilitate the participants during the pre-recorded classes, pausing, and resuming the video at various points throughout the class (non-continuous, no interaction with instructor). Among all the different modes of participation there was a clear preference for participating in-person:

I would probably say that the activity center is the most fun. To come down to the (activity) center and dance rather than having to stand at home in the kitchen alone.

*I think the very best thing is when we are together, because then you also see what the others are doing and can perhaps learn something from them too. Just when you think ‘this is impossible for me’, I think that at the same time you can also learn how others manage this. And I also think it’s really nice that we’re together (…). At home it is not the same at all.*

There was a clear preference for participating in-person, and especially for the monthly sessions when an instructor was present in-person:

*Twice I have been there with instructors physically present. It’s absolutely the most fun. And I seem to be “on” when the instructor is on. It’s also better than streaming from YouTube.*

*It was definitely much nicer to be there physically. It means a lot that there is a living person in front of you. And with whom you can have a little eye contact once in a while, and (an instructor) who also sees us and notices that we may have to do that turn 4 or 5 more times before everyone is on board.*

Those who participated at via pre-recorded classes found the instruction (especially the salsa) repetitive and boring at times:

“*We didn’t get that far in salsa either. I felt the same and the same steps we kept going on and on and on*.”

“*I don’t think I’m getting as much out of it as I expected (…). We expected more that we got to learn to dance a little salsa, and those steps, it’s repetition, repetition, repetition… But we never really learned how to start salsa dancing*.”

The main obstacles to participation were busyness, prior commitments to other activities, lack of enthusiasm for dancing alone, and illness.

“*It’s not something I would want to do in the future, because I have so many other activities that it’s kind of squeezed in. So, I don’t think I will choose it in the future*.”

### Challenges

Within this main theme, five subthemes were identified: technical difficulties, challenges with their own movement, challenges with the instruction, self-consciousness, and challenges with the method. Many participants reported frustration and difficulty following the instruction due to technical difficulties. This was not the fault of the participants, but due to technical difficulties associated with audio streaming and transmitting over live-stream.

“*Sometimes the music was too loud and other times it was too low. There were also some technical things that were a bit tedious*.”

“*The difficulties with online instruction might be that there is a little bit of a delay… so that the sound and image are not completely followed. (The instructor) also mentioned at one point, when she looked at us, that ‘it’s as if you don’t quite follow the rhythm.’ And I think it is simply that sound and image were not synchronized. So, there is a slight shift, which might make it a bit difficult to follow along, especially if you have difficulty hearing the rhythm in the music.*

Participants also reported challenges with their own movement during the instruction, due to self-consciousness or physical difficulties:

“*I had problems with balance. First, I didn’t want to go backwards. When it went too fast and the others moved too fast, I got dizzy. I tried to move, but I got dizzy very often*.”

Participants also reported that they overcame these challenges as they grew more accustomed to the intervention:

“*The challenge was that in the beginning, you had to learn to free yourself and your body and everything. Until you thought, ‘I can probably also do it, if the others can.’ So, that has probably been the biggest challenge - to free oneself*.”

Participants describe a wide range of experiences, which resulted in different challenges with regards to the instruction. For some, the level was too easy and boring, whereas others found the instruction too fast-paced. Some participants did not feel they got much out of the lessons because they perceived the level of instruction was not matched to their ability:

“*Salsa has been a challenge for me because it is very different from what I usually dance and it is also pure rhythm, but it is very different from (what I’m used to). But it’s exciting to try something different and I’d like to continue with that, too*.”

“*Well, I was also expecting us to get a bit more dancing out of it, instead of it being the same and the same and the same. And it was basic and basic and basic all the tim*e.“

Participants who attended centers with streaming of pre-recorded classes expressed the most difficulties:

*” I think maybe also the difference for us who (had pre-recorded classes), from those who had the live-streamed (classes), was that there you could ask some questions (…). We didn’t really have the opportunity to do that because it wasn’t live*.”

“*It was a little frustrating to sit and look at something like that. It was streamed, and we could see that she had a dialogue with someone who was not us. So, it was then, what to say, it was not particularly conducive, I think*.”

Although the intervention lasted 12 weeks, the study ran over a period of seven months, which meant the participants had different starting and completion times. While this allowed for greater flexibility in recruiting, several participants found it frustrating because it limited their perceived progress in learning the dance forms, due to the varying experience levels of the new people joining the study:

“*Because we started at many different times: some were tested for the first time already in November, and I joined in January… There were many of the others who were much further along than we were. I think that was perhaps a bit of a disadvantage and a bit of a chore for them too, because it was repetition for them*.”

“*I came in after the project had already been running. So, the first couple of times I think they were a bit difficult, but then things gradually got better with those salsa steps*.”

### Progression

Within this main theme, two subthemes were identified: physical progression and skill transference. Most participants were previously familiar with salsa dance but were new to improvisation and were reluctant at first. The main reasons for initial discomfort were (1) being observed by others (reluctance to turn cameras on, or be seen by people passing by, and (2) unfamiliarity with the lack of rhythmic movements, which is a feature of improvised dance. However, many participants reported that their attitudes towards improvised dance changed over time, and they grew to appreciate the freedom of improvisation, which they eventually found challenging and fun.

“*I actually came to like (free dance) a lot, and I could feel that the body liked it; I think this is a good development*.”

Participants also appreciated the opportunity to move with their entire bodies, not only focusing on hips and footwork. Many participants reported a progression in their comfort level and ability with improvisation dance during the 12-week intervention.

“*We were always asked (by the dance instructor) if we had got our heart rate up, and at least I don’t think I did at first. But then it’s like the more you go there, and the more times you dance with the others, the pulse starts to come up, so we know where to start the exercise*.”

“*It was very ‘strange’ for me until I got used to it. But fun. And… it is true that you get hold of some other muscles than you otherwise think you use*.”

Overall, participants were less likely to feel they had progressed with learning salsa, either because they had already been attending salsa classes prior to the study, or because they didn’t have the opportunity to learn several dance styles:

“*I love salsa, but the two different dance styles are two very different dances if you can put it that way. And I haven’t developed that much in the salsa area.“*

Some participants noted that the two different styles were complementary, and could see a good connection and transference of skills from one style to the other:

“*I think it was cool that you did something to loosen up your body. And then we used it to loosen up the body on Thursday and danced the combination of the two (salsa and free dance). I think is really good. … I can dance myself, but I can also see that my body needs the other, so I think the combination is great*.”

### Motivation

Within this main theme, three subthemes were identified: (1) social connection, (2) involvement in research study, and (3) interest in dancing. Participants were motivated in part by the opportunity to be together in a group, and the social dimensions and opportunities that were created around the dance sessions held at the activity center. Participants reported that the social connection was a major motivating factor for their participation in the study, and their overall enjoyment:

“*There is also a social aspect to being together and I certainly wanted that too.“*

“*I would say half of it was the social interaction, so you get to meet people you know, and (whom) I have also gone to yoga with. It’s always nice - that togetherness*.”

In general, most participants preferred dancing together in a joint location than dancing alone at home:

“*I’ve tried to (dance) at home a few times, I guess it wasn’t very fun, because there wasn’t any laughter, there wasn’t anyone to talk to. (…). So, I like best that it was with others. To have that community and that laugh, and just the small talk you get at the same time. That’s why I’ve had the best time getting out among others and doing that*.”

“*Especially when you’re alone like I am, it’s good to get out and be social with others. I use my activities for that, that is primarily you can say the social, and then it’s so nice that you get to use your body in a good way at the same time. But it’s mainly the social thing I’m after*.”

Participation in a research study was a motivating factor for some participants, who were interested in contributing to a research study, get opportunities to learn something new, and were keen to find out their individual results:

“*If you sign up for something, you also complete it, at least that’s how I feel. Otherwise, it’s a waste of people’s time*.”

“*I want to continue until it’s over and it’s like to see how far we can really go with dance and learn something*.”

General interest in dancing and enthusiasm for learning new dance styles was a motivating factor for many participants:

“*It was an opportunity to be allowed to dance, because otherwise I wouldn’t have joined. But then, I train balance and strength training every day, and I walk a lot. So, I move quite a lot, and then I think there is no music (in those other activities). So, this was a welcome gift to me*.”

“*I thought it could be very exciting to see what could come of it. And us older people, we like to move. Besides swimming and aqua-gymnastics and yoga, this could also come in*.”

Despite that only women completed the study, the focus groups did not discuss whether the group makeup influenced their enjoyment, motivation, or participation in the program.

### Perceived health and wellbeing

Within this main theme, three subthemes were identified: (1) physical changes, (2) social, emotional, and mental health, and (3) connection to everyday life. Some participants found the movement challenging, but the majority found the dance instruction very enjoyable. Many responded that they noticed physical changes in their own health and wellbeing, especially concerning balance, improved posture, muscle strength, greater flexibility, and reduced lower back pain:

“*I could actually feel in my body that it wasn’t so crooked at all even. Because I got it after all; I could feel my whole body, it was somehow activated*.”

“*I don’t think I had been (dancing) many times before I could feel that I was more flexible in my body, and I can also feel that I have gained more muscle strength in my legs.*“

A few participants reported that the intervention caused them pain and soreness, from using muscles they were not accustomed to using:

“*(I noticed feelings of) well-being and a little bit of soreness because that was something I didn’t have (before). …I haven’t tried to activate so many small muscles in my body. But you hit them. And it was only positive.*“

“*I’ve gotten better at standing on one leg. (I know this) because I go to yoga, and there I couldn’t keep my balance for very long, and it’s better now. (…) Sometimes had to stand on one leg during the dance, and I could actually do that for a long time, whereas I had problems with it in yoga. So that’s why I think it’s because of the dance’s that it’s gotten better*.”

*”I think it actually gave me a sense of well-being, and it gave me some good, and some tips on how to stay flexible*.”

“*In the first few times I think I was in pain. Now, I have osteoporosis, and I think I had pain in my back. But after quite a few times, it has loosened up somehow. And I love those different movements, it does something really good for my body, I can feel it. (…) you become much more flexible, and especially your classes there, I love them. Because you get the whole body, legs, arms, and everything moving, and it does something really good. And I would also say balance, I can feel that too. Yeah, standing and putting on socks and all that stuff now is a lot easier than it was before I started this project*.”

“*I really just enjoyed getting my upper body going. I can feel that my movements around the salsa day, they have really changed in the period that has passed, in fact in a relatively short time. There is a different movement in the upper body because of salsa, and also because we sat on a chair (in free dance) and really used the body for the warm-up (…)*.”

“*So, the flexibility thing, I actually notice that after three times or something like that. Muscle strength, I can see that, some fat has disappeared on the legs and muscles have come instead, and I can feel it too. The demanding exercise that I do for yoga is much easier now than before*.”

“*I definitely think I can feel in my balance, that it has improved. (Although) I haven’t had much trouble with the balance, but it has improved*.”

“*I think I have gained greater body awareness and a straighter back. And you can say that I feel more grounded. I have also gotten better at feeling my feet in the ground. And like the others, I think that I have become more agile and flexible in my body. It also feels like the ability to balance has improved. … I notice that when I go to yoga, where we do balance exercises. I have become better at keeping my balance on one leg in different postures for longer than before*.”

Other participants were less sure that the perceived changes were a result of the dance training, because of their already active lifestyles:

“*I also go to several things, and it can be difficult to distinguish which is which. (…) I always take the stairs rather than the lift, and I’m not someone who goes for long walks, but I move a lot in everyday life. But I have an awfully big house with stairs up and stairs down. (…) I would like to be able to say, it’s your dance, it’s (the instructor’s) dance that has done it, but I can’t say that. I don’t know, unfortunately.*“

“*It is difficult to decide, because we have so much other training that has fallen in the same period. So, I can’t separate that. I have definitely improved in both balance and flexibility*.”

Only a few participants reported experiencing no change at all:

“*I don’t feel that I have become physically stronger…. I do so much in advance and train so much balance and fitness on the side. But it has of course been different and exciting to be here, but I don’t think it has helped physically*.”

“*I can’t feel any difference because I’m already used to moving a lot both in the swimming pool and when I walk. I can’t feel that it has gotten better or worse or anything else. So, it’s been a good experience, it’s physical; I can’t feel any difference there*.”

Several participants reported making new acquaintances and friendships as a result of the intervention, and increased social activities, such as arranging to meet for coffee or lunch after the dance classes:

“*But it has been nice to be with someone. When we finished, we were such a bunch. Who could go in and have a good time and have a cup of coffee and talk afterwards. And you wouldn’t have done that if you were sitting alone at home*.”

“*It’s been good (…) and there have also been people who don’t usually come to the center but want to continue coming here. I think that is a good place to come*.”

Some participants became inspired to self-organize group activities for online streaming of other classes:

“*I live in community (housing), so at half past five I have invited my roommates over to dance in the communal house. So now we do that once a week, and it’s somewhat inspired by the fact that we danced online down at the center*.”

Those participating at home had fewer opportunities for social engagement. Predictably, responses to perceived changes in social and emotional health differed based on whether the participants followed instruction at the activity center or at home. For those who danced both in-person and online, they all preferred social aspects on dancing in-person with others. One participant who only participated online said:

“*When I’ve danced, I’ve danced via YouTube and others haven’t been on that. And since I’ve pretty much always run most of the sessions on YouTube. So, when I’ve been online and I’ve danced online, I was kind of attuned to it. So, I don’t know if I missed being able to look at someone*.”

Several participants reported noticing the benefits of the dance in other areas of their everyday life, including activities of daily living (ADL):

“*Dancing makes you more flexible. Then it becomes easier when you have dropped something, and you must go down after it and wash windows and things like that. So it is, as we like to say, both hands and feet, so that you get a lighter body in one way or another, a flexible body – it’s great*.”

“*I think the body is lighter in one way or another. And you find it easier to do the different things. It can be both cleaning, it can be vacuuming, it can be anything. I think the body has become lighter, and not so stiff in the lim*b.“

“*I have had many good stretches there, and balance. But I haven’t improved in balance either, at least in the test. I can’t understand that. But I feel I have a better balance. For example, I think I’ve gotten better at relaxing, because when you’re afraid of falling, you tense up. I think I’ve gotten better at relaxing. (…) So there has been improved balance, I am absolutely sure of that*.”

### Online dance

Within this main theme, six subthemes were identified: familiarity/enjoying freedom of movement, connection to instructors, lack of feedback, flexibility of YouTube, individual effort, suggestions for improvement. Participants were reluctant to have cameras on initially, and it took some convincing from the instructors to put on their cameras (if at home), and others who participated at the activity centers experienced feelings of self-consciousness with the unfamiliar movements (especially in free dance):

“*It may well be that I run a little wild sometimes during (salsa). Because maybe it went too slow or too fast. But it was great with you, you could choose for yourself. There wasn’t such a firm frame.*“

Many reported that they became less inhibited over time and learned to enjoy the free movement.

“*I think I learned to be more free. I wasn’t as inhibited when we finished dancing as I was before we started*.”

Participants reported that they mostly felt connected to the instructors, although they would have wished for in-person and more personalised instruction:

“*I also think that I ‘know’ both (instructors) because you become close in one way or another. We do silly things with (them), and (they) do the same with us.“*

Participants noted that while online instruction was different than the presence of live instructors, the instructors accommodated their needs and established a relationship with them:

“*It cannot be compared to being together and the instructor also being physically present. And the thing about dancing together. But I’ve been really happy with the online instruction, and I really think I’ve gotten good instructions from both (instructors)*.”

*”I think it has been perfectly okay to follow along online. I felt connected to what was happening on the screen*.”

“*As far as I’m concerned, I don’t think it matters that much. Of course, it’s nice to have someone physically there, but when it can’t be otherwise, I think it’s fine*.”

Several participants mentioned lack of feedback on their dance steps from the instructors as a main criticism of the project and online dance instruction, with several participants mentioning that they specifically wanted to “be corrected” when they had done something wrong. This was especially pronounced among participants who participated at activity centers that chose to stream pre-recorded videos (YouTube), where there was no opportunity for the instructors to give personalised feedback or motivation to individuals:

“*That’s probably what I miss the most: it’s that you have to be corrected and you can’t do that very well when, for example, (the instructor) is standing in front of the screen at home and we’re standing in a room. After all, you can’t stand around shouting ‘now you have to do such and such.’ Otherwise, I think it’s fantastic that it can be realized with such an online program, it’s fantastic.“*

Online dancing was preferable for some, as it addressed some of their challenges:

“*Online, that’s the advantage, of course you can do it from home, so you don’t have to spend time on transport, and the disadvantage is simply that the community isn’t there. And the fact that you can learn a little from each other even when you are standing there and are a little more active. At least that’s how I think. It’s such immediate advantages and disadvantages.“*

“*The advantage of it being online is that we can go to YouTube when we get home and then we can try to practice a little more if it’s something we just can’t figure out, and then rewind it. We can’t do that when you’re physically present. So, you can’t go home and say to him, how did we do there? We can do that when you have it on YouTube, that’s an advantage*.”

“*One of the things that attracted me when I signed up for (the study) was that it was online (…) After all I live far away and have to drive for it. And it’s fantastic that you can just press a few buttons and still be involved*.”

Several participants who attended both in-person at activity centers and at home reported differences in their perceived effort, depending on where they participated:

*”I think I get more out of it if I make more of an effort when I’m with others. At home I like to go and cheat a little*.”

“*I think the difference is that when I am with others and see others, it is easier to get other ideas than to follow the verbal instructions that you have to give. It becomes a little less imaginative when I have to use my own movement patterns and cannot be inspired by others*.”

“*I’ve been streaming it at home, so I’ve been alone and it’s a very big difference, and not nearly as rewarding, and it’s been hard for me to keep myself engaged dancing around at home. So, it is clearly the social aspect that plays a role and the attendance which is important: it is what gives joy that you have the others and the persistence as well*.”

## Discussion

The main findings of this exploratory study were that several indices of functional fitness in older women improved over time after taking part in a 12-week online, community-implemented dance intervention consisting of salsa and improvision, whereas the participants’ feelings of loneliness did not appear to change. Although the quantitative measures for loneliness and HRQOL did not reveal a significant change, the qualitative results from the post-intervention interviews suggest that the intervention had an overall positive impact on health and wellbeing, especially the social engagement. Specifically, the qualitative results suggest that when implemented in a community setting with mutual participation by others, online dancing may positively influence social engagement and the older women’s perception of their own physical abilities as well as mental and emotional wellbeing. Interestingly, the older women’s self-perception of enhanced physical abilities and emotional wellbeing was observed in the qualitative data, but not in the surveys, which should be considered when designing future studies to evaluate wellbeing and HRQOL.

The discrepancy in the results from the quantitative and qualitative data concerning wellbeing and HRQOL evidence the need for more mixed-methods research into the impact of dance and other complex interventions. A limited number of mixed-method studies have explored the impact of dance training on mental and physical health in older adults. Ambegoankar et al. reported that twice weekly ballroom dance sessions for ten weeks had a positive impact on cognition and physical health determined by the Short Physical Performance Battery [7]. In another study, O’Toole et al. showed that one weekly session for six weeks of creative expression and contemporary dance routines for older adults enhanced community and social participation as well as the participants perception of their own emotional well-being [11]. Taken together, the findings from our study are consistent with the findings from previous investigations suggesting that dance training may provide a strategy for engaging older adults in physical activity and support mental and physical health outcomes. A novel contribution of this study is the demonstration of the feasibility of an online, group dance intervention that contributed to positive benefits in physical health and wellbeing, including social connection as well as significant improvements in functional fitness. However, our findings also show there were differences in the experiences of those who participated with live-streaming instruction and those who participated with pre-recorded instruction, due to the lack of interaction and feedback on specific dance steps when dancing to the pre-recorded instruction videos.

Online instruction implies that participants can follow the dance training via a computer at their homes (i.e., remotely), which may directly address frequently reported physical activity barriers for older adults, including transportation issues, time constraints, and inclement weather [6], while at the same time reducing the need for public space requirements and physical presence by the instructors. Therefore, when considering the reported health effects from dancing combined with the practical advantages of remote instruction, online interventions could potentially provide one possible solution for mitigating the increasing economical public healthcare burdens associated with the still growing older adult population.

We identified a few challenges with the online group dance training. These challenges primarily concerned sound and streaming, namely the ability of the participants to hear both the music and the vocal instructions given by the dance instructors. There was no trouble with connectivity. The research team was made aware of the sound issue early on, and the sound issues were resolved within the first three weeks that the study ran. Another challenge identified within the Online Dancing theme (*Subtheme 6.3: Lack of Feedback*). The instructors only found out at a very advanced point in the study period that some of the participants were streaming the lessons regularly outside of the live instruction dance times. This was not how the intervention was intended, so the research team had limited ability to control, or course correct for this.

### Physical health

#### Functional fitness

As the majority of risk factors for chronic diseases increase with age, the adoption of regular physical activity is crucial to buffer the decline in physiological reserve of organ systems associated with aging [34] and to improve mental and physical health outcomes among older adults. However, maintaining a certain level of physiological capacity during aging is not only important for attenuating chronic disease risk but also for retaining the ability to perform ADL independently. Using the SFT, we observed significant increases over time in several aspects of fitness, including upper body strength (arm curls), aerobic endurance (2-min step test), and hamstring flexibility (chair sit-and-reach). Such improvements in fitness were generally supported by the qualitative results where many participants responded that they had noticed positive physical changes in their own health, especially concerning balance, muscle strength, improved posture, and greater flexibility. That in-person dance training can improve flexibility, muscle strength, and aerobic endurance in older women is congruent with the existing literature [35, 36]. In a systematic review and meta-analysis of 29 randomized clinical trials (all but one trial included mainly women), Mattle et al. reported benefits of in-person dance-based mind-motor activities on several aspects of fitness, including balance, mobility, and muscle strength [35]. In another systematic review including 18 studies using various dance styles, Hwang et al. reported that dancing, regardless of style, generally seem to improve measures of aerobic fitness and muscle strength in older adults when delivered in-person [36]. Overall, the results from these systematic reviews are complemented by the findings from the current study and suggest that online dancing may be considered a beneficial method for improving several aspects of functional fitness concurrently, while at the same time allowing for flexibility in where and when to exercise and for delivering this opportunity to more people than would be financially feasible with in-person instructions. Considering that older adults frequently report time constraints as a reason for not being physically active [6], reducing the time-commitment that could otherwise occur in in-person training settings (i.e., strength training, aerobic training, and flexibility at a fitness center) could be of great value. Such perspectives were also supported by the qualitative results as several of the participants mentioned that they have busy schedules that include various responsibilities such as physical activities and social engagements.

The significant and large effect size for the pre-post change in chair sit-and-reach performance indicates that the dance training was associated with profound demands to hamstring flexibility suggesting that weekly online dance sessions, throughout 12 weeks, improve hamstring flexibility in older women. Consistent with the quantitative results, results from the interviews confirmed that several of the participants felt that they were more flexible after 12 weeks of dancing.

The significant increase in the number of arm curls performed in our study may seem surprising as a recent meta-analysis did not show any significant effect of dance training on arm curls test performance in older adults [37]. A possible reason for improvement in the arm curl test after 12 weeks could be enhancement of neuromuscular recruitment and coordination in the upper limb. Indeed, when older adults experienced in dancing are compared with matched controls without dancing experience, better multi-muscle coordination and synergism were found in the dancing group [38]. However, it is important to note that the findings presented by Wang et al. [38]. was observed in the lower limb, while the present results show improvement in the upper arm.

Finally, results showed a significant increase in 2-min step test performance, indicative of improved aerobic endurance. While we did not measure cardiorespiratory fitness directly, the 2-min step test has shown to correlate significantly with other more direct measures of cardiorespiratory fitness suggesting that the 2-min step test can be used a valid field-based surrogate measure of cardiorespiratory fitness [25]. Not only is retainment of a certain level of fitness required for maintaining the ability to perform ADL, such as climbing the stairs, mounting evidence also suggests that low levels of cardiorespiratory fitness may be an even stronger predictor of mortality than established cardiovascular disease risk factors such as smoking, high cholesterol, hypertension, and type 2 diabetes mellitus [39]. Accordingly, dance classes might be a way to maintain functional independence and reducing overall mortality risk through enhancement or preservation of cardiorespiratory fitness.

#### Body mass and composition

An interesting observation was the significant increase in body mass (and BMI) over time. However, such gross measures of body composition are insensitive for distinguishing between changes in fat and lean body mass. Indeed, the increase in body mass was not accompanied by similar changes in surrogate measures of body fat and distribution (i.e., sum of skin folds and waist circumference). In fact, waist circumference, a valid surrogate measure of visceral adiposity, was, if anything, lower at the post-intervention assessment (mean ∆ from baseline: -1.3 cm, *P* = 0.051). Considering such almost significant reduction in waist circumference, despite increases in body mass, could indicate that the dance training may have led to accretion in lean mass, which was also supported by the qualitative results, where several participants reported experiencing increased muscle strength over the study period. However, direct measurements of body composition by magnetic resonance imaging or dual energy X-ray absorptiometry scans are needed to test this hypothesis.

### Mental health and wellbeing

#### Questionnaires

Although the change over time in TLS, as a measure of loneliness, almost reached statistical significance, the small effect size indicate that such change may not have been clinically meaningful. The small effect could relate to the fact that none of the participants were feeling lonely at baseline. In fact, at baseline all the participants could be categorized as having either a ‘low’ or ‘moderate’ degree of loneliness according to Perry et al. [32], leaving little room for improvement. This was supported by the qualitative results in which most of the participants responded that they were already extremely social before the study began and involved in several ongoing activities. Notably, there were, however, three participants less at the post-intervention who reported a moderate degree of loneliness (and instead reported a low degree of loneliness), suggesting a positive change in mental health for these participants. Similarly, there were no changes in any of the SF-36 summary scores suggesting that the intervention did not impact either mental or physical components of HRQOL. Again, the explanation may relate to the generally healthy cohort of older women included in the study, as the participants at baseline already reported their HRQOL to be at the high end of the summary scores. Nonetheless, evidence of positive changes in wellbeing was found in the qualitative results, wherein several participants responded during the interviews that the dance training resulted in joyful social activities (“*It gives such joy to be here with others”)*, and a different self-consciousness about their own body, which made them feel “freer” and happier.

#### Participants’ experiences of the intervention

In general, most participants found the dance instruction very enjoyable and self-reported noticeable positive changes in both their physical health and wellbeing, including perceived improvements in flexibility and balance, improved posture, muscle strength, greater flexibility, and reduced lower back pain. Moreover, participants were mostly satisfied with the level and quality of online instruction. However, there was a significant difference in the experiences of those who participated with live-streaming instruction, and those who participated with pre-recorded instruction. One important factor from the qualitative results was that several participants who attended both at activity centers and at home reported differences in their perceived effort and motivation, depending on where they participated. Participants were more likely to remain physically and mentally engaged if they were participating in a group at an activity center than if they were at home alone.

Several participants mentioned lack of feedback on their dance steps from the instructors as a main criticism of the online dance instruction. This was especially pronounced among participants who took part at activity centers that chose to stream pre-recorded videos, where there was no opportunity for the instructors to give personalized feedback or motivation to individuals. Many participants struggled with this one-sided dimension. This is not normally a problem that is encountered during in-person or live-streamed dance classes and should be addressed in future studies with online dance training. On the other hand, some participants with more familiarity with technology emphasized the flexibility of online dancing from YouTube as preferable, because it could be done on their own time and did not require parking or other hassles.

### Strengths and limitations

A major strength of this exploratory study is the use of both qualitative (focus group interviews) and quantitative (blood pressure, body composition, SFT, and questionnaires) measures, which allowed us to explore the benefits of online dance training on health from a holistic perspective, i.e., assessment of both mental and physical health outcomes with multiple instruments. Additionally, the qualitative data for perceived physical health and wellbeing revealed benefits of the intervention that would not have been captured if relying on quantitative instruments alone (loneliness and HRQOL questionnaires). Such observations emphasize the merits of mixed-method research when evaluating complex health interventions. Another noticeable strength is the high degree of ecologicall validity in the study, as the intervention was implemented in practice, thereby closely approximating the environment that we aim to generalize our findings to.

Nonetheless, the study does have some limitations. The lack of a control group is a limitation, as we are unable to compare the changes in the intervention group against natural time-course changes over 12 weeks for this population, or quantify the inherent variability in outcome measures, including a potential “learning effect” from the repeated testing. Similarly, no restrictions were made regarding participation in other sports or training activities during the study period. While this may have enhanced the ecological validity of the study, it may also limit the ability to distinguish the benefits of the intervention from other activities.

Consistent with the majority of previous studies [35, 36], our results might not be generalizable to older men, as all but one of the participants were women, with the only man dropping out due to feeling uncomfortable with being outnumbered. The overwhelmingly female bias is consistent with the literature on dance interventions, and sex-related barriers in recruitment and retention should be addressed in future studies.

The current study is limited by the lack of control of participant attendance, which may negatively affect the accuracy of objectively assessing the effectiveness of the intervention. By design, participants were allowed to play (and replay) the dance classes from YouTube on their own, and thus we were unable to accurately record participant attendance. Accordingly, some participants may have attended the classes more than twice weekly, while others may have attended only once weekly, resulting in uncertainty in the exact frequency of training and thus total volume of dancing during the intervention period. While this was a limitation, it also approximated a more realistic, real-life context for our study. Future studies may consider applying a stricter control of participant attendance, or alternatively instruct participants to log the number of attended classes. In this way, it may also be possible to evaluate a potential dose-response relationship between the number of weekly classes and the change in health outcomes. Also, we did not control or monitor the exercise intensity during the dance classes, which is a limitation as we are unable to report the exact physiological stimuli that the participants were exposed to.

A common risk of physical activity interventions conducted with older adults is high dropout rates [40]. A high dropout rate was also evident in our study, as 13 out of 45 participants (29%) dropped out of the study before the final post-intervention assessment. Although not all dropouts were study-related, a dropout rate of almost 30% should be considered when evaluating the viability of online dance interventions among older adults. As a result of the high dropout rate and the lack of intention-to-treat analysis, our results may be somewhat biased towards those who completed the intervention and found the intervention enjoyable. To address this limitation, we did invite all participants for the post-intervention interview, so those participants who dropped out could share their perspectives and have a more comprehensive and balanced reflection of both the positive and negative aspects of the intervention. Unfortunately, none of those participants who dropped out were able to attend the interview. Finally, as the dropout was higher and the effect size lower than expected, the study was underpowered to detect a significant change in loneliness (obtained power = 50%).

### Barriers for future implementation

Important insights and experiences were obtained from this exploratory study that may help to improve future implementation of online dance training interventions in community settings. Specifically, we identified several barriers that should be addressed prior to successful implementation:

- Adequate time should be devoted prior to the intervention to resolve any technical difficulties associated with audio streaming and transmitting over live-stream.
- While pre-recorded online dance classes offer flexibility in terms of when and where to attend, the lack of personalized feedback on dancing technique and one-sided communication from the instructor may negative impact the participants enjoyment and motivation to attend.
- Participant retention and enjoyment may potentially be improved with live-instruction, allowing for a more personalized instruction and feedback and instructor-participant interaction. Even intermittent contact with a live instructor might improve participants’ overall experience.

## Conclusion

This exploratory mixed-methods study showed that 12 weeks of community-implemented online dance training improved several aspects of physical health among older women, including aerobic endurance, upper body strength, and hamstring flexibility, as well as the older women’s self-perception of their own improved physical abilities, self-efficacy, and wellbeing. While most participants found the online intervention enjoyable and socially rewarding, several participants missed the feedback from the instructors that otherwise would occur with in-person instruction. The results speak to participants’ self-perception of increased flexibility and range and ease of movement, as well as increased social activities as a result of the online intervention. Further research with stricter control of study variables is warranted to further investigate the efficacy of online dance training on physical and mental health in older adults. Finally, given the multifactorial risk of fall accidents among older adults, future research may explore the benefits of online dance training on fall-related risk factors, such as gait speed and postural control, and other positive changes in behavior.

## Data Availability

The datasets used and analyzed in the presented study are available from the corresponding author on reasonable request.
